# Anterograde Gastroduodenal Intussusception: A Rare but Lethal Complication of Percutaneous Endoscopic Gastrostomy Tube Placement

**DOI:** 10.7759/cureus.4347

**Published:** 2019-03-30

**Authors:** Mohammad Alomari, Ahmed Alomari, Asif Hitawala, Shrouq Khazaaleh, Laith A Al Momani

**Affiliations:** 1 Internal Medicine, Cleveland Clinic Foundation, Cleveland, USA; 2 Internal Medicine, The Hashmite University, Al-Zarqa, JOR; 3 Internal Medicine, Cleveland Clinic - Fairview Hospital, Cleveland , USA; 4 Internal Medicine, East Tennessee State University, Johnson City, USA

**Keywords:** peg, complications of peg, adult intussusception, gastric outlet obstruction

## Abstract

Percutaneous endoscopic gastrostomy (PEG) tube placement is one of the methods of providing enteral nutrition support and is often used in critically ill patients. There are several complications of PEG tube placement, including intussusception. Jejunojejunal and retrograde jejunoduodenogastric intussusception are well-documented complications of PEG tube placement. Here we describe the case of a 25-year-old female who was diagnosed with anterograde gastroduodenal intussusception with the PEG tube acting as a lead point. Our case is unique as, to the best of our knowledge, there are no documented cases of PEG tube-related anterograde gastroduodenal intussusception. The reported patient was found to have extensive gastric pneumatosis and portal venous gas concerning for acute ischemia. Such cases warrant immediate surgical intervention. However, in our case, the patient’s family opted for comfort care measures.

## Introduction

Anterograde gastroduodenal intussusception is the rarest form of adult intussusception accounting for less than 10% of all intussusception cases in adults [1]. This rare presentation has been documented to be caused by pedunculated polyps [2], Menetrier’s disease [3], hamartomas [4], gastrointestinal stromal tumors [5], and other gastric tumors. It is thought that luminal lesions act as a lead point for the anterograde prolapse of the gastric wall into the proximal duodenum [6].

Percutaneous endoscopic gastrostomy (PEG) tube is the modality of choice for providing enteral access to patients who require long-term enteral support. Although generally considered safe, PEG tube placement can be associated with many potential complications, including, but not limited to, intestinal trauma, hepato-splenic injury and gastro-colo-cutaneous fistula [7]. Moreover, gastroduodenal intussusception may rarely occur with associated substantial morbidity and mortality [8].

Although no formal consensus for the management of gastroduodenal intussusception does exist, it is widely accepted that removal of the lesion acting as a lead point and subsequent resection of the necrotic bowel are the mainstay of treatment [6]. We herein present a rare but life-threatening complication from indwelling PEG tube. Our findings emphasize the importance of early clinical diagnosis in order to guide timely management.

## Case presentation

A 25-year-old female was hospitalized with generalized abdominal pain, low-grade fever, rigors, lethargy, and vomiting. Her medical history was significant for Down syndrome and Moya Moya disease complicated by multiple strokes and intracranial hemorrhage requiring an external ventricular drain and subsequent PEG tube placement for enteral support one month prior to presentation.

Vital signs on admission showed sinus tachycardia at 130 bpm, a temperature of 101 F and blood pressure of 110/65 mmHg. Abdominal examination revealed a distended, diffusely tender abdomen with evidence of bloody brownish exudate at the ostomy site. Pertinent laboratory studies included: elevated white blood cell count of 21 × 103 /μL (normal 4 to 11 x 103 /µL), hemoglobin of 9 gm/dL- which was relatively decreased from a previous value of 12 gm/dL (normal 11.5-15.5 gm/dL), metabolic acidosis with pH of 7.25 (normal 7.35-7.45) and elevated serum lactate of 7.27 mmol/L (normal 0.5-1 mmol/L).

The patient was transferred to the medical intensive care unit for suspected sepsis and was resuscitated with intravenous normal saline and broad-spectrum antibiotics. A few hours later, she started to have coffee ground emesis with a subsequent drop in her blood pressure.

**Figure 1:**
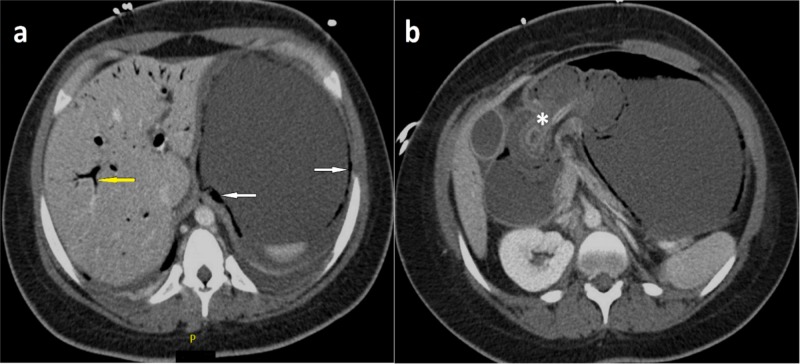
Axial plane computed tomography of the abdomen and pelvis (with contrast) at two different levels (T12: a and L1: b) showing percutaneous endoscopic gastrostomy tube displacement into the proximal duodenum (asterisk) with evidence of portal vein gas (yellow arrow) and pneumatosis (white arrows).

**Figure 2:**
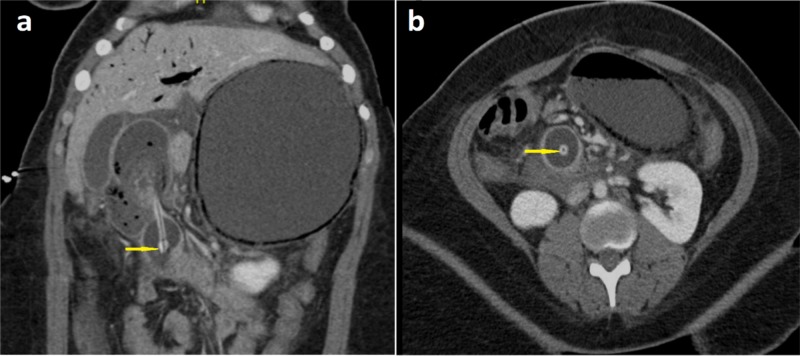
Coronal and axial plane computed tomography (a and b, respectively) of the abdomen and pelvis (with contrast) showing percutaneous endoscopic gastrostomy tube tip acting as a lead point at the point of caliber change (yellow arrows).

The abdominal plain radiograph showed a dilated stomach. This was followed up by an abdominal computed tomography (CT) scan with intravenous contrast demonstrating a dilated distal esophagus and stomach with extensive stomach wall pneumatosis and portal venous gas concerning for acute ischemia, the PEG tube was displaced into the proximal duodenum (Figure 1a-1b) with its tip at the point of caliber change (Figure 2a-2b). Distal to the PEG tube tip, the third duodenum and the remaining small bowel were collapsed. There was no evidence of pneumoperitoneum.

The patient was diagnosed with iatrogenic anterograde gastroduodenal intussusception with the PEG tube acting as a lead point with complicating small bowel necrosis. Family members were counseled regarding the need for immediate surgical intervention. However, the patient’s family opted for comfort care measures. No surgical intervention was done and the patient was transferred under hospice care.

## Discussion

Since endoscopic insertion of a gastrostomy tube was first introduced in 1980 by Gauderer et al. [9], it has increasingly become the method of choice to obtain long-term gastric access. Approximately 10% of the institutionalized elderly depend on PEG tubes for feeding [10], with an annual estimate of 100,000 to 125,000 PEG tubes being placed in the United States [11]. To date, little attention has been paid towards PEG tube-related gastric outlet obstruction as a serious but preventable complication.

Previous case reports described retrograde jejunogastric intussusception causing small bowel obstruction due to PEG tubes, as the tube can migrate forward into the jejunum and act as the lead point [8]. Although the underlying mechanism of intussusception is not entirely understood, it has been hypothesized that if the external bolster on the gastrostomy tube was not properly fixed, it might allow for the tube to migrate away from the abdominal wall and to slide forward through the gastrostomy tract into the duodenum aided by the propelling forces of normal peristalsis. Further peristalsis then telescopes the stomach antrum into the duodenum, thus setting the stage for an antegrade intussusception [12]. Hence, during placement of the PEG tube, it is important that the positioning disc is secured to the skin and the tube be secured to the positioning disc. If the gastrostomy tube is not anchored appropriately, there is a risk of migration, intussusception, and necrosis of the small bowel [8]. In our case, it is believed that the PEG tube may not have been anchored securely enough, giving rise to this complication.

There are a few case reports of PEG tube related intussusception. Pelosof et al. [13] reported a case of retrograde jejunogastric intussusception due to PEG tube where the PEG tube was put in place without any external fixation device. Wu TH et al. [14] published a case report with jejunojejunal intussusception following jejunostomy in which they mention possible mechanisms of intussusception as retrograde peristalsis of jejunum during vomiting and injecting force produced by tube feeding with pump infusion on the jejunostomy tube, which acts as a stent. To the best of our knowledge, our case is the first reported case in the adult population describing PEG tube related anterograde gastroduodenal intussusception where the feeding tube acted as the lead point.

CT is a useful and reliable investigation in making a preoperative diagnosis, especially in giving anatomic details of the intussusceptum, intussuscipiens, and the adjacent organs [15]. The characteristic features include an inhomogeneous “target” or “sausage”- shaped soft- tissue mass with a layering effect: mesenteric vessels within the bowel lumen are also typical. A CT scan may define the location, the nature of the mass, its relationship to surrounding tissues, and additionally, it may aid with staging suspected malignancies that might have caused the intussusception [6]. In our case, the CT scan clearly demonstrated the displaced PEG tube in addition to signs of acute ischemia and collapse of the small bowel distal to the PEG tube.

Successful management of intussusception depends on early diagnosis, adequate resuscitation, and prompt reduction. Due to a significant risk of associated malignancy, radiologic decompression is not addressed preoperatively in adults [16]. Therefore, 70%-90% of adult cases of intussusception require definite treatment, of which surgical resection is, most often, the treatment of choice [6]. In our case, urgent surgical intervention was warranted due to associated necrosis and ischemia of the small bowel. Unfortunately, our patient had significant comorbidities in addition to having a late presentation with complications. After a detailed discussion, the family opted against any surgical or curative intervention and chose to transfer the patient to hospice care.

## Conclusions

Although PEG tube-related mechanical complications are not uncommon, presentation in the form of gastroduodenal intussusception is exceptionally rare. This diagnosis should always be entertained in a patient with acute gastric outlet obstruction and unexplained clinical deterioration. Ensuring proper fixation of the PEG tube to prevent distal migration may help avoid this complication. The treatment in adults usually requires surgical intervention with resection of the involved bowel segment.
